# Journal Of Cardiovascular Disease Research CURRENT EDITORIAL TEAM - 2010

**DOI:** 10.4103/0975-3583.59977

**Published:** 2010

## International Consulting Editor of JCDR


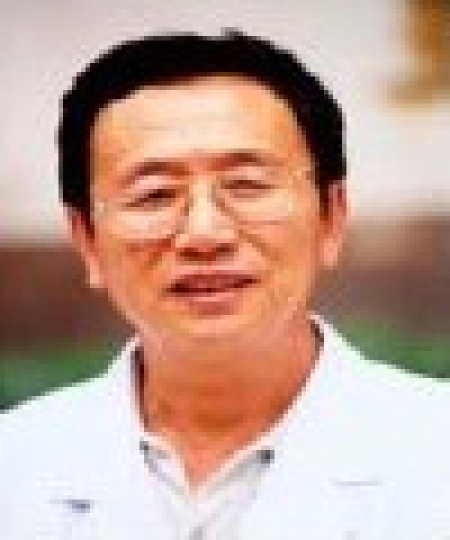
 Dayi Hu, MD, FACC, FESC, current Chief of Heart Center, Peking University People’s Hospital; Dean of Clinical Research Institute, School of Public Health, Shanghai Fudan University; President of Chinese Society of Cardiology (CSC); President of Chinese College of Cardiovascular Physicians (CCCP). Professor Hu is a pioneer of radio-frequency ablation technology and evidence-based medicine in China. He has specialized in preventive cardiology, electrophysiology and interventional therapy of coronary artery disease. He is also engaged in the treatment and prevention of hypertension, coronary heart disease, heart failure and ionic channelopathy. He is the principal investigator (PI) of China Heart Survey, and the Steer Committee member and PI of many international clinical trials such as ROCKET, BEAUTIFUL, SHIFIT, ACE, MAGELLaN, etc. Professor Hu is the Editor-in-chief of Chinese Journal of Cardiology, international board member of Nature Cardiovascular Medicine, Journal of Clinical Cardiology, Journal of the American College of Cardiology, European Heart Journal.

## Editor-in-Chief


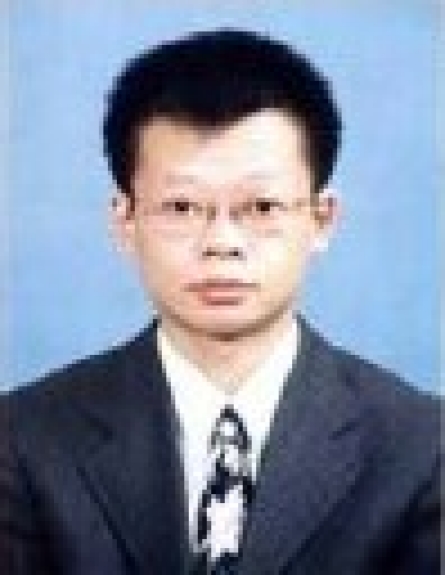
 Dr. PengZhou, M.D.& Ph.D, Cardiologist. Presently, he serves as a research fellow in Cardiology, Wake Forest University School of Medicine, USA. More than 15 years career in Cardiology, He has authored over 60 publications, contributed over 4 book chapters. He specializes in both basic research and clinical practice with emphasis on ionic channel function, signal transduction, heart failure and interventional therapy of arrhythmia and CAD. Since 2007, 11 of his first author and co-author research achievements have been invited to present in AHA Scientific Session.

## Associate Editor


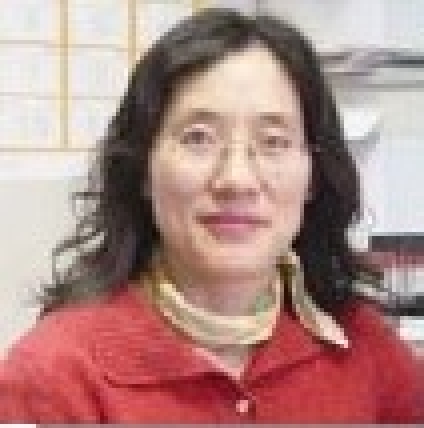
 Cuilan Li, Ph.D, associate professor is now working in Cardiology Department, Peking University People’s Hospital, Beijing, China, as a Senior Research Scientist and Chief of Basic Research Lab of the Department. She has engaged in the basic research of cardiac electrophysiology for 20 years and published about 80 papers. She is currently involved in the projects on gene screening of long QT syndrome and other channelopathies. She is an international recognized research scientist and in charge of the National Channelopathy Registry in China.

## Associate Editor


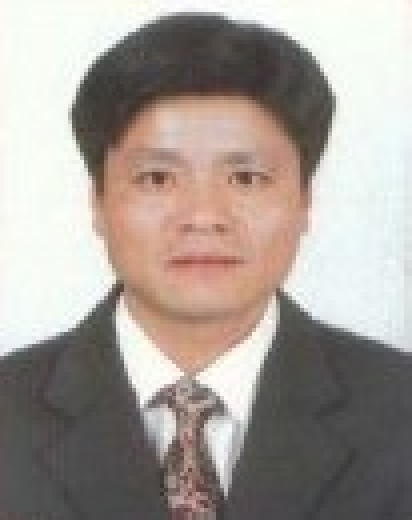
 Yujie Zhu, Ph.D. a Senior Member of IEEE, is a recognized scientist in cardiac electrophysiology and biomedical engineering. He is currently working in Division of Cardiovascular Disease, University of Alabama at Birmingham, USA. He serves as a reviewer for several prestigious international cardiovascular journals. He received several awards. He has authored and coauthored numerous papers. Dr. Zhu’s research interests include electrophysiological properties of cardiac ion channels, mechanisms of arrhythmia, and application of biomedical engineering in cardiovascular diseases.

## Associate Editor


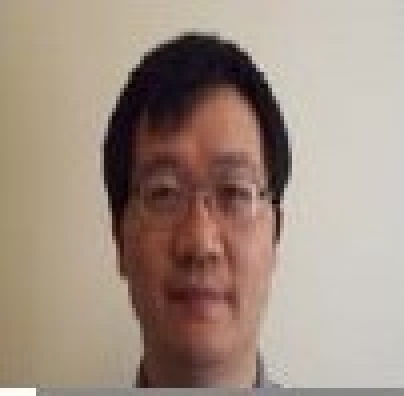
 Zhi Xu, M.D.& Ph.D., Research Associate at the W.M. Keck Center for Transgene Research, University of Notre Dame. Dr. Xu specializes in exploring the roles of PAI-1 in the pathophysiology of atherosclerosis and heart fibrosis. He published in some of most prestigious journals, including *Blood, JBC* and *Journal of Thrombosis and Haemostasis*. As a PI, His research has been supported by AHA. Additionally, he serves as an ad hoc reviewer for a number of journals such as *Blood, Current Drug Targets*.

## Publishing Editor


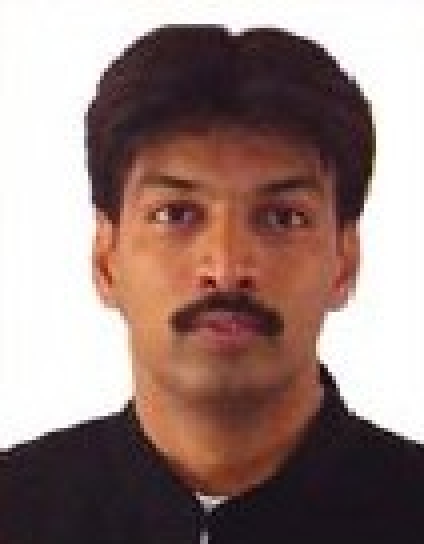
 Mueen Ahmed K. K. Ph.D, Assistant Professor at College of Clinical Pharmacy, King Faisal University, Al-Hassa, KSA. His major research interest has been screening of natural products for pharmacological activity with special emphasis on cardiovascular activity. He has published many papers in peer-reviewed international journals. Presently, Dr. Ahmed is involved and working in several editorial boards of international circulating professional journals, serving as editor for Journals such as *Pharmacognosy Research*, associate editor of *Pharmacognosy Magazine, Pharmacognosy Reviews etc*.

## Assistant Editor


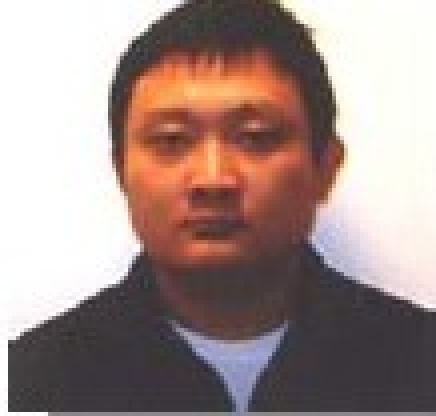
 Ningpu Yu, Ph.D. is a research scientist in NIH, Bethesda, Maryland, USA. Dr. Yu has eight years experience in studying the molecular and cellular mechanisms of cardiovascular inflammation and discovery of potential drug targets and therapies for atherosclerosis and diabetes. He was invited to present his work at the AHA Scientific Sessions multiple times. Dr. Yu has served as reviewer for professional journals and government grants.

## Assistant Editor


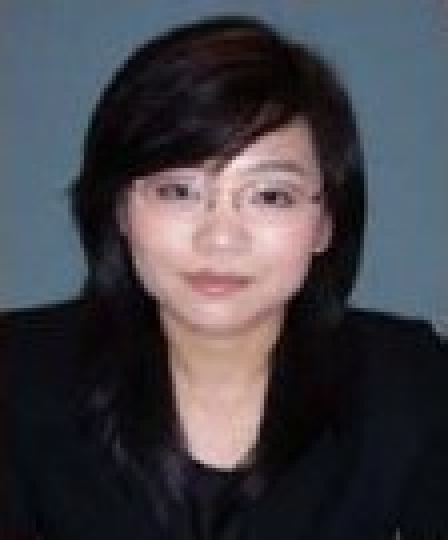
 Dr. Ping Li, M.D. Ph.D., is now working in Department of Cardiology, Nagoya University School of Medicine, Japan, as a research fellow. She has engaged in the clinical and research work in Cardiology for nearly 10 years. Her researches are mainly involved in cardiovascular regeneration. She has published about 20 papers.

## Editorial Board Member


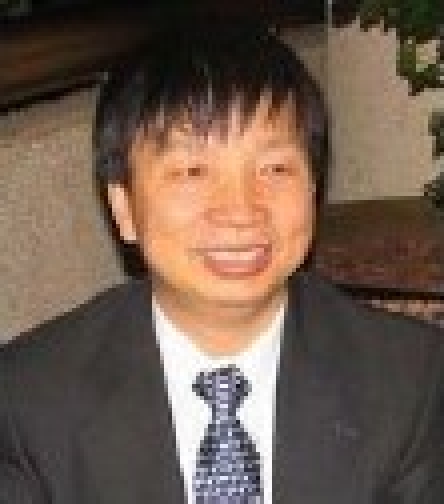
 Xin-Chun Yang, M.D. and Ph.D., Professor. Director of Heart Center, Beijing Chaoyang Hospital, affiliated Capital Medical University, Vice-Dean of the Department of Cardiovascular Diseases, Capital Medical University. He is quite accomplished in the field of interventional cardiology and has made prominent contributions in the non-pharmacological treatment of arrhythmia. Presently, professor Yang is presiding over many research programs such as National Natural Science Foundation project and Eleventh Five-Year Science and Technology Support sub-Program etc.

## Editorial Board Member


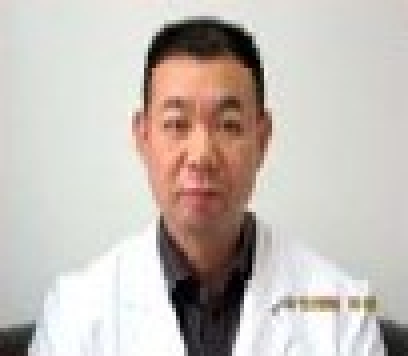
 Dr. Dong Ran, M.D.& M.S, Vice-Dean of Department of Cardiac Surgery, Anzhen Hospital. Having been engaged in the career of cardiovascular surgery for more than 20 years, he has authored over 20 publications, and co-authored 3 book chapters. Presently, he serves as an editorial member of *Journal of Cardiovascular and Pulmonary Diseases*, which was founded by Dr. Wu Yingkai, the pioneer of heart surgery in China. These works enabled him the ability to focus on controversial topic of cardiac techniques.

## Editorial Board Member


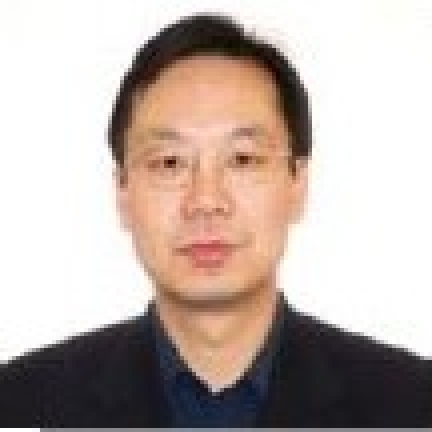
 Jianjun Zhang, M.D.& Ph.D., Professor of Medicine, Director, Section on Cardiology of West Campus of Affiliated Chaoyang Hospital of Capital University of Medical Science. Prof. Zhang is accomplished in electrophysiology and interventional therapy. He is a current council member for Beijing Division of Chinese Medical Association. Presently he serves as editorial board member for *Chinese Journal of Cardiac Pacing* and *Electrophysiology and Chinese Journal for clinicians.*

## Editorial Board Member


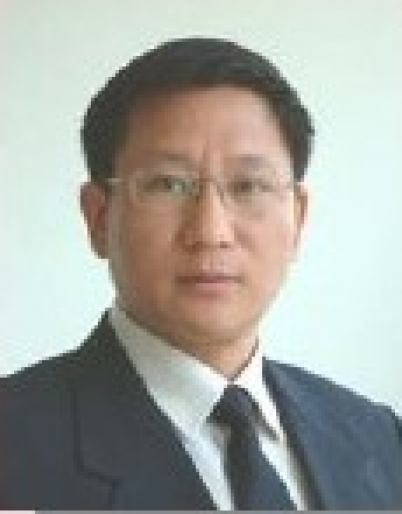
 Jun-Hua Wang, M.D. and Ph.D., Cardiologist, Vice-Director of the Department of Cardiology, Air Force General Hospital, Beijing, China. In his 20 years Cardiology practice, he published about 30 articles in peer-reviewed, national or international professional journals. He possesses ample experience in diagnosis and treatment of cardiovascular disease with emphasis on interventional therapy of arrhythmia and coronary artery disease. As the Principle Investigator of Air Force General Hospital branch, Dr. Wang had taken part in ONTARGET/TRANSCEND, an international, large scale, multi-center evidence-based medicine study.

## Editorial Board Member


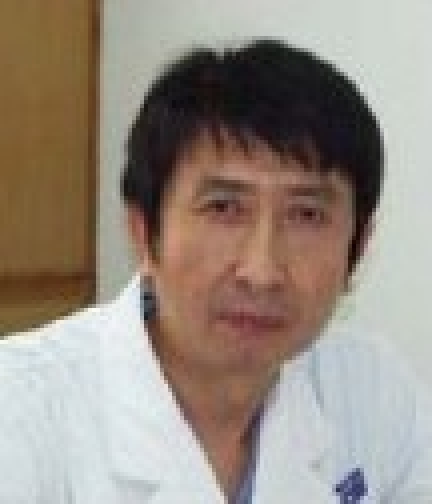
 Dr. Ren Wenlin, M.D.& M.M, cardiac chief physician, presently, he serves as the chief in Cardiovascular Center, Beijing Chuiyangliu Hospital, Beijing, P.R.China. More than 26 years clinical practice in cardiology, he has authored over 30 publications, and contributed over 6 book chapters.

## Editorial Board Member


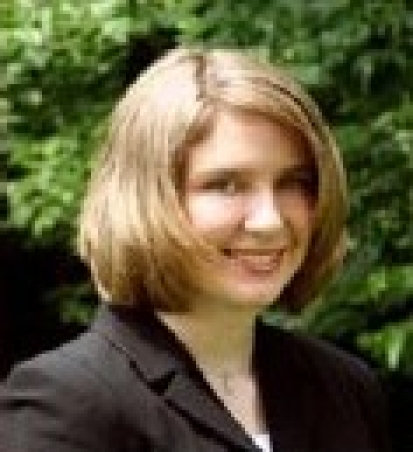
 Elizabeth Gilbert, Ph.D., Postdoctoral Research Associate in the Department of Animal and Poultry Sciences at Virginia Tech. Her research interests include comparative nutrition, protein and amino acid metabolism, nutrient transporters, type 2 diabetes and pancreatic beta cell regeneration, flavonoids as nutraceuticals, cell proliferation and apoptosis signaling pathways. Dr. Elizabeth Gilbert was one of the recipients of the 2009 Milton L. Sunde Award from the American Society for Nutrition for outstanding experimental, applied or fundamental research in nutrition that uses an avian species.

## Editorial Board Member


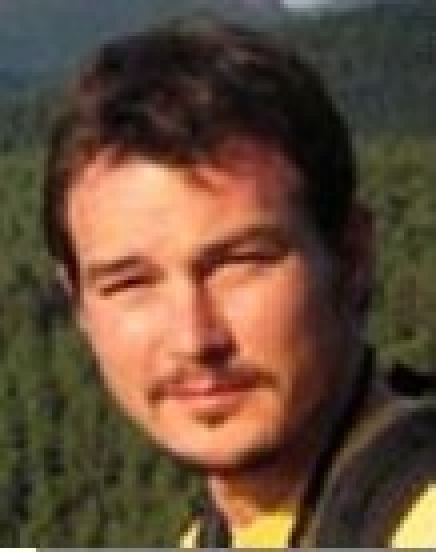
 Cedric Viero, Ph.D, Research Associate of Department of Cardiology, Wales Heart Research Institute, School of Medicine, Cardiff University, UK. Dr. Cedric Viero specializes in ion channel, calcium signaling and cardiac electrophysiology. He is a current member of the Working Group on Cardiac Cellular Electrophysiology, European Society of Cardiology. In his cardiovascular basic research career, he has authored more than 20 publications. Recently, he served as editor for several peer-reviewed, international professional journals.

## Editorial Board Member


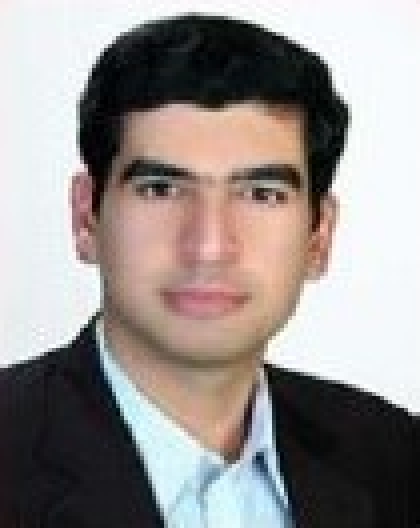
 Amir Aslani, M.D, Cardiologist, Presently, he serves as an interventional electrophysiologist in Section on Cardiology, Shiraz University School of Medicine, Iran. He has authored over 50 publications. He has been selected as the best young investigator in 13^th^ Razi Festival. Recently, he has approved as a “Senior Scientist” by “*Nature*” journal.

## Editorial Board Member


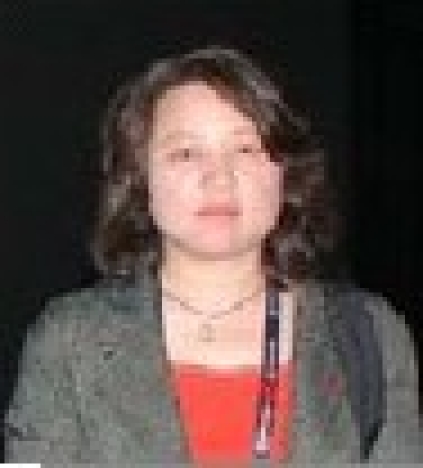
 Guang-Zhen Zhong, M.D.& Ph.D, associate professor, working (clinical, teaching, and scientific research) in Beijing Chao-Yang Hospital, Capital Medical University, has been engaged in Cardiology for about 12 years. She published several papers on “J Hypert” “Life Sci”, “Heart and vessels”, “Int J Cardio” and so on. She gave an oral presentation on AHA Scientific Symposium in 2008. She achieved quite a few grants from government.

## Editorial Board Member


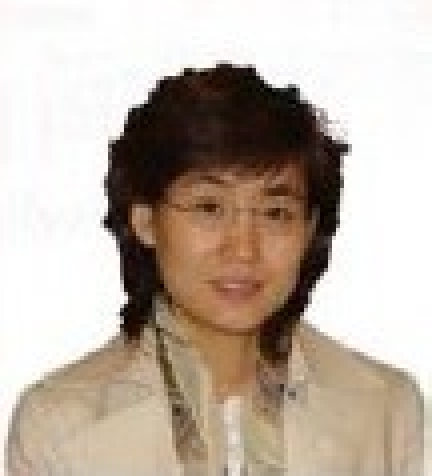
 Yihong Sun, M.D, Associated Professor of Medicine in the Heart Center at Peking University People’s Hospital. Her research interests are prevention of cardiovascular disease, antithrombotic and antiplatelet therapy, cardiovascular epidemiology and large clinical trials. She is currently working with the ACE trial in Oxford University to investigate acarbose therapy in the prevention of cardiovascular events in patients with impaired glucose tolerance and established cardiovascular disease.

## Editorial Board Member


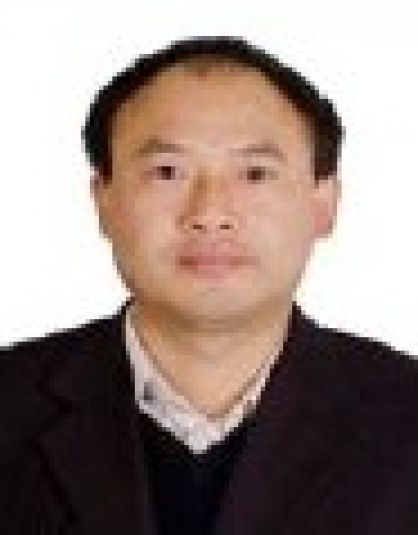
 Zhang ChaoJun, M.D., Ph.D.Associate Professor, Department of General Surgery, Division of Thyroid, Mammary& Vascular Surgery Xinqiao Hospital, Third Military Medical University, Chongqing, China PRC. Dr. Zhang is a recognized general surgery expert in the field and accomplished in vascular surgery with emphasis on peripheral vascular diseases, such as abdominal aortic aneurysm, complicated peripheral arterial aneurysm, atherosclerosis. More than 12 years vascular surgery career he has been involved in about 2000 vascular cases.

## Editorial Board Member


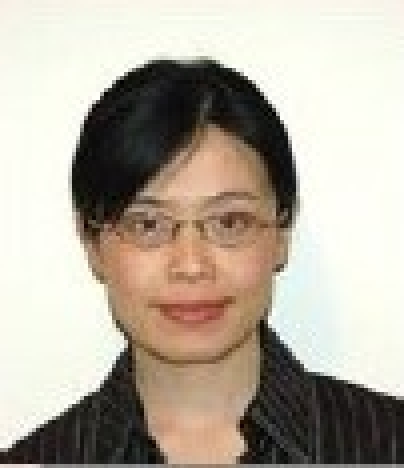
 Yongxia Sarah Qu, MD, PhD, is an Assistant Professor at the Department of Medicine/ Cardiology, State University of New York, Downstate Medical Center. Dr Qu is involved in both patient care and basic translational cardiovascular research. Dr Qu published over 30 peerreviewed papers in high impact journals. She has been funded for her research by both NIH and the Department of Veterans Affairs. Dr Qu has contributed significantly to the research of autoimmune-associated congenital heart block and has interest in channelopathies.

## Editorial Board Member


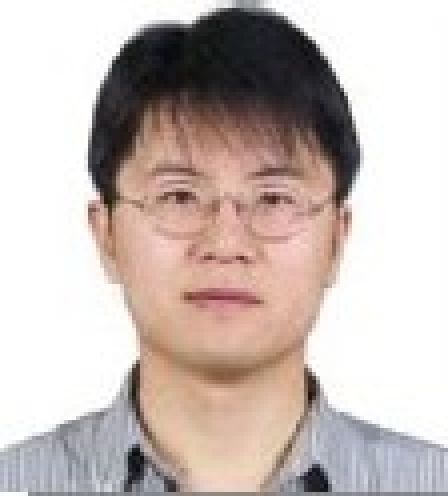
 Dr. Guang Yang, M.D.& Ph.D, currently serves as postdoctoral fellow in Vascular Biology Center in Medical College of Georgia, Augusta, Georgia USA. He also serves as reviewers for several professional journals. Dr. Yang’s medical background, including two years of clinical experience, together with 8 years genetic and physiological studies, has contributed to the cardiovascular biology research. In the last 10 years, Dr. Yang has authored over 20 publications and presented his works in international meetings.

## Editorial Board Member


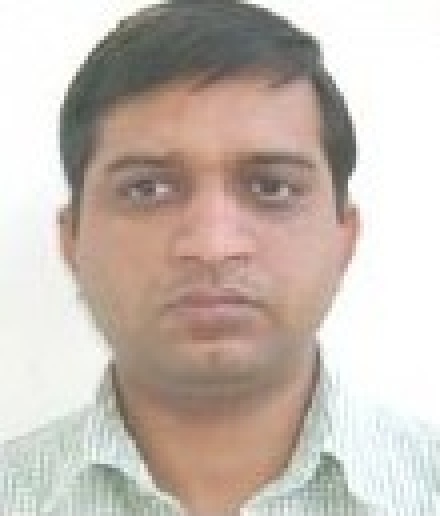
 Alok Kumar Singh, M.D& DM, Senior resident in cardiology, in CHATRAPATI SAHU JI MAHARAJ MEDICAL UNIVERSITY Lucknow India. More than 6 year in cardiology, He has about authored 20 publications and contributed 4 book chapters. He had been appointed as assistant editor of journal of clinical and diagnostic research for 2010. He has special interest in interventional cardiology and coronary artery disease, heart failure and rheumatic heart disease.

## Editorial Board Member


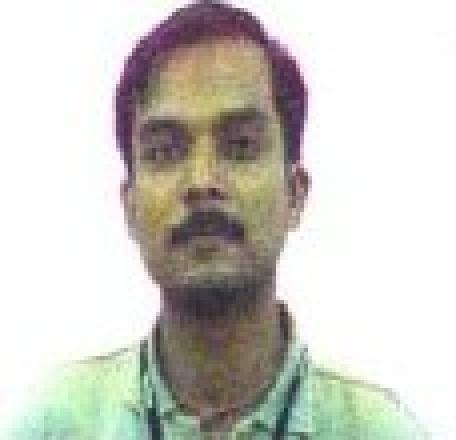
 Debmalya Barh, M.Sc., M.Tech., M.Phill., PhD. Consultant Biotechnologist and presently serves as Honorary Scientist, IIOAB, India. First to work on Cardiac Myxoma signaling pathway and drug targets. He has authored over 20 international publications with first authorship and contributed over 5 book chapters during 2008–2009. He also serves as editorial and review board members of many professional journals.

## Editorial Board Member


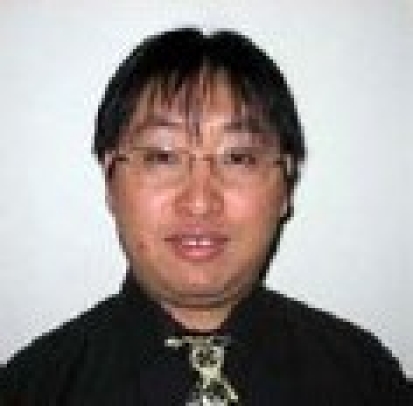
 Dr. Xianming Zhang, Ph.D., a research associate in Department of Pharmacology at University of Illinois at Chicago, has engaged research in experimental medicine about ten years and authorized over 20 papers in prestigious professional journals. His current research focuses on exploring the roles of kallikrein-kinin and rennin-angiotensin system in cardiovascular pathophysiology by investigating interaction among proteins of both systems. Recently Dr. Zhang was awarded fellowship by American Heart Association due to his series of interesting findings.

## Editorial Board Member


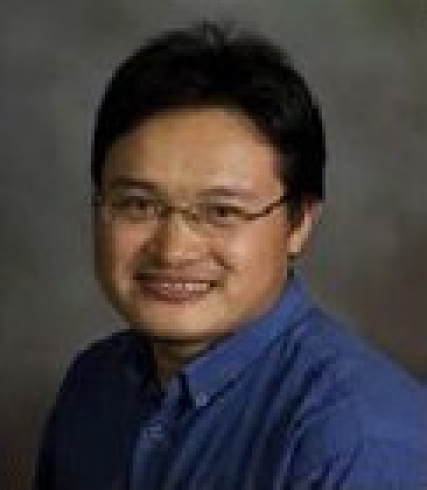
 Hongwei Si, Ph.D., postdoctoral research associate in the Department of Human Nutrition, Foods and Exercise at Virginia Polytechnic Institute and State University (Virginia Tech), USA. His research focuses on the beneficial effects and mechanisms of food-derived polyphenols on cardiovascular diseases, diabetes, obesity and aging. He has authored over 20 publications. Currently, he serves as editorial members, reviewers for numerous prestigious journals.

## Editorial Board Member


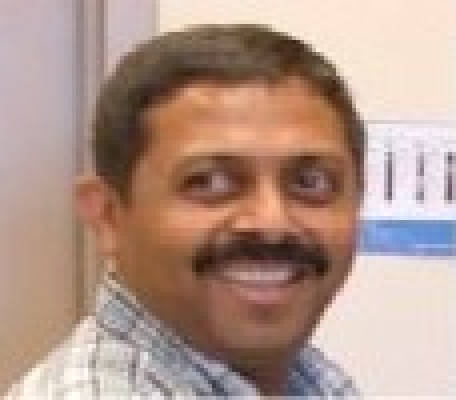
 Dr. Bishwanath Chatterjee is presently a Staff Scientist at National Heart Lung and Blood Institute and is an expert in genetics and genomics of congenital and adult heart disease.

## Editorial Board Member


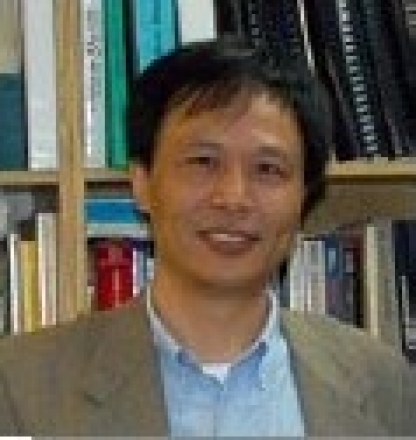
 Dr. Cheng Wang, Ph.D., associate professor of biomedical engineering at Shanghai Jiao Tong University. He is currently the vice-chairman of the Department of Biomedical Engineering at the School of Medicine. His research interests have longtime focused on the signal acquisition and automated processing of the electrocardiogram. In recent years, he concentrates his research effort on the image-based quantitative evaluation of the myocardium function.

## Editorial Board Member


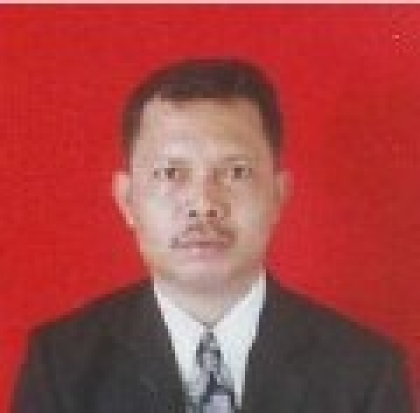
 Yohanes Buang, PhD, attending vessel blood maintenance and the regulation of metabolism disorders. He has authored many publication related to the disorders of metabolism. He also serves as a reviewer for some international professional journals. Recently, he focuses his work on vessel blood chemicals and the disorders generated on Department of Chemistry, Faculty of Science and Engineering, Nusa Cendana University, Indonesia.

## Editorial Board Member


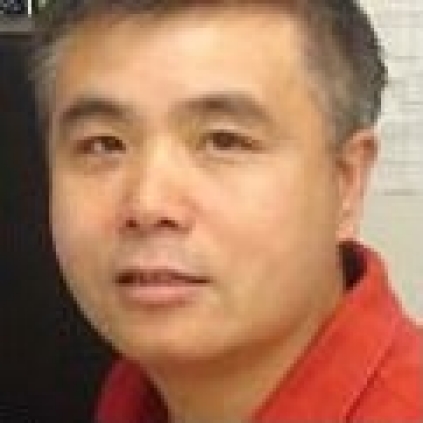
 Jibin Zhou, M.D.& Ph.D. Dr. Zhou’s focused on the regulation of normal and stress-induced cardiomyocyte hypertrophy. Specifically, he studies the signal transduction pathways that regulate growth responses, focusing on protein kinase cascades. He has studied the role of glycogen synthase kinase-3 alpha and beta. Additionally, he has recently identified a novel mechanism by which oxidant stress may signal cell death via a SOK1/14-3-3zeta/ASK1 cascade.

## Editorial Board Member


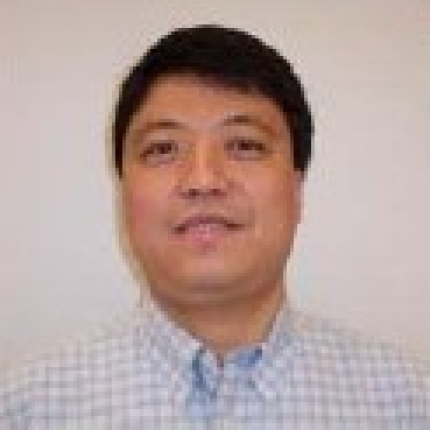
 Kai Shen, Ph.D. in Chemistry. He is currently a research associate in the Department of Biochemistry and Biophysics of the University of North Carolina at Chapel Hill. With research expertise across the areas of hypertension, molecular cardiology and vascular medicine, his publications extend from biochemistry to nanotechnology and have been cited over 100 times. He also serves as a reviewer and member of editorial board for professional journals.

## Editorial Board Member


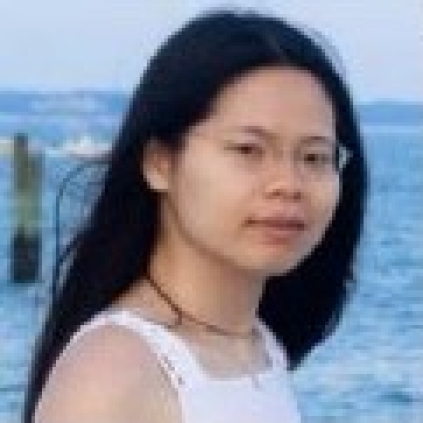
 Xuan Gao (Biophysics, Boston University) Her Ph.D. work is to determine effects of curvature, protein composition and oxidation on structural stability and functional remodeling of human plasma HDL. These works were published in 3 peer reviewed papers, and presented in 9 international conferences. She was invited for platform talks by OPTM (2006, 2008) and Protein Society (2009). She is recipient of a Young Investigator Award from Kern Aspen Lipid Conference.

## Editorial Board Member


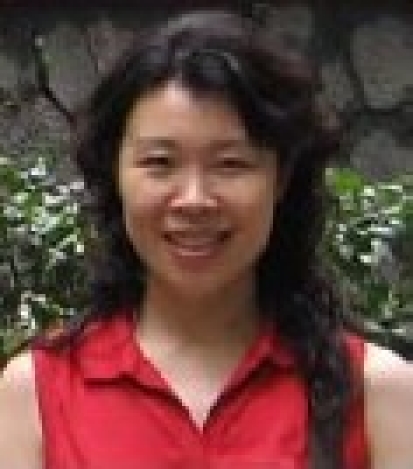
 Fang Liu, M.D. Associate Chief Physician, Department of Geriatric Cardiology, Peking University First Hospital. Dr. Liu specializes in clinical cardiology and echocardiogram. Recently, she serves as an editorial member of *Chinese Journal of Multiple Organ Diseases in the Elderly*. She is Echo-Specialist of Peking Physician Association and Director of Bejing Institute of Ultrasound in Medicine

